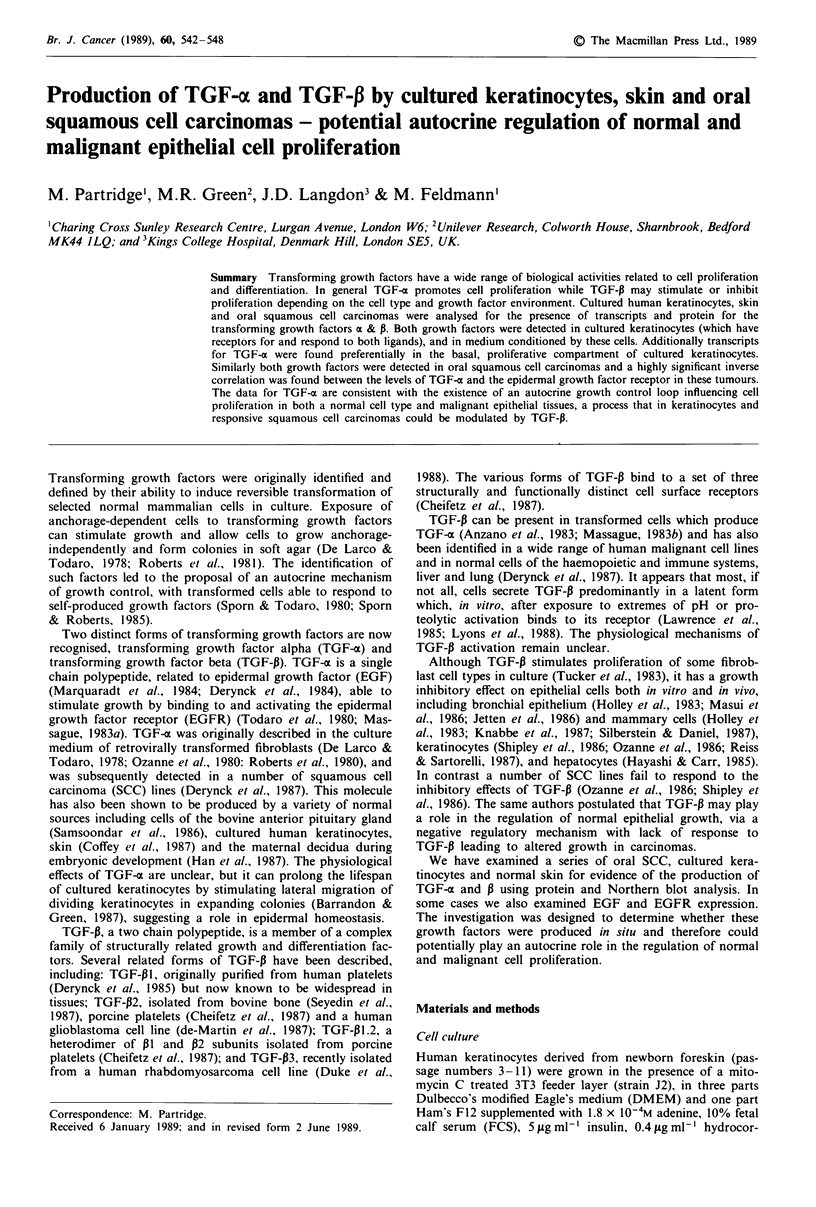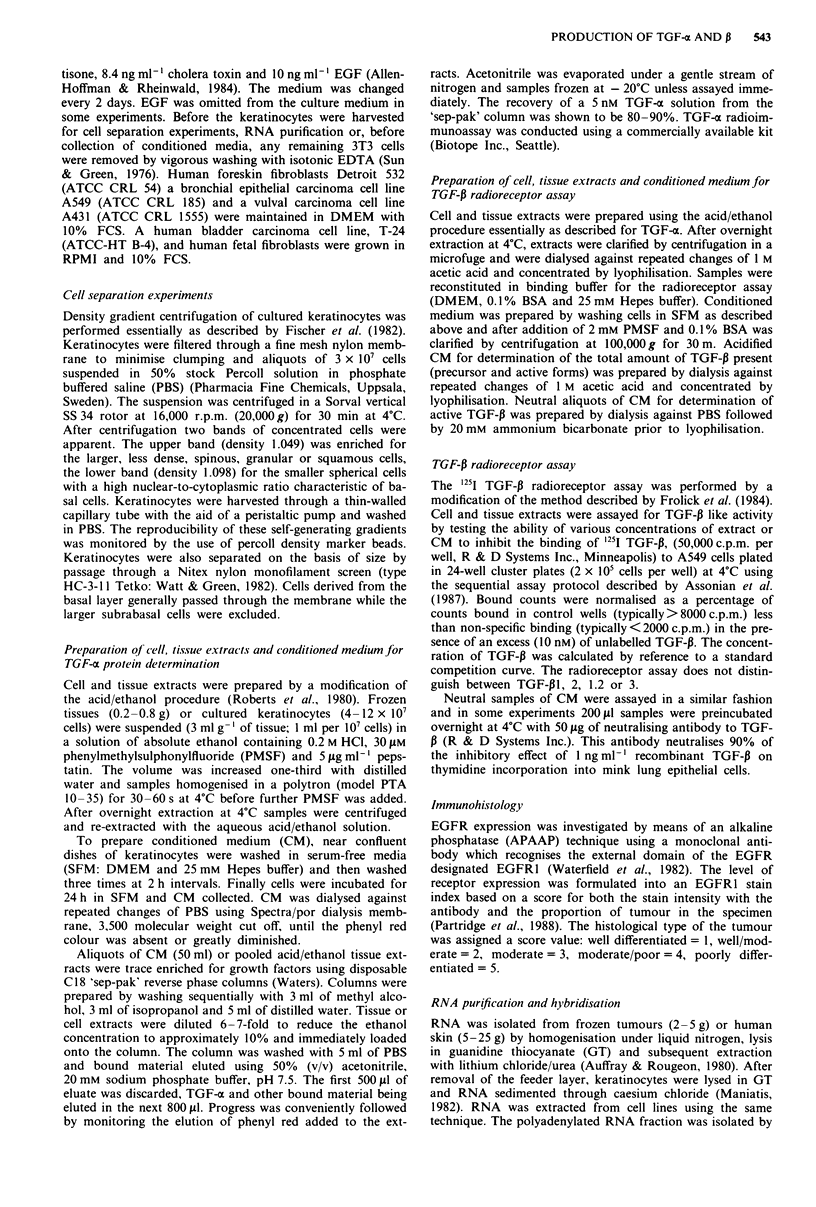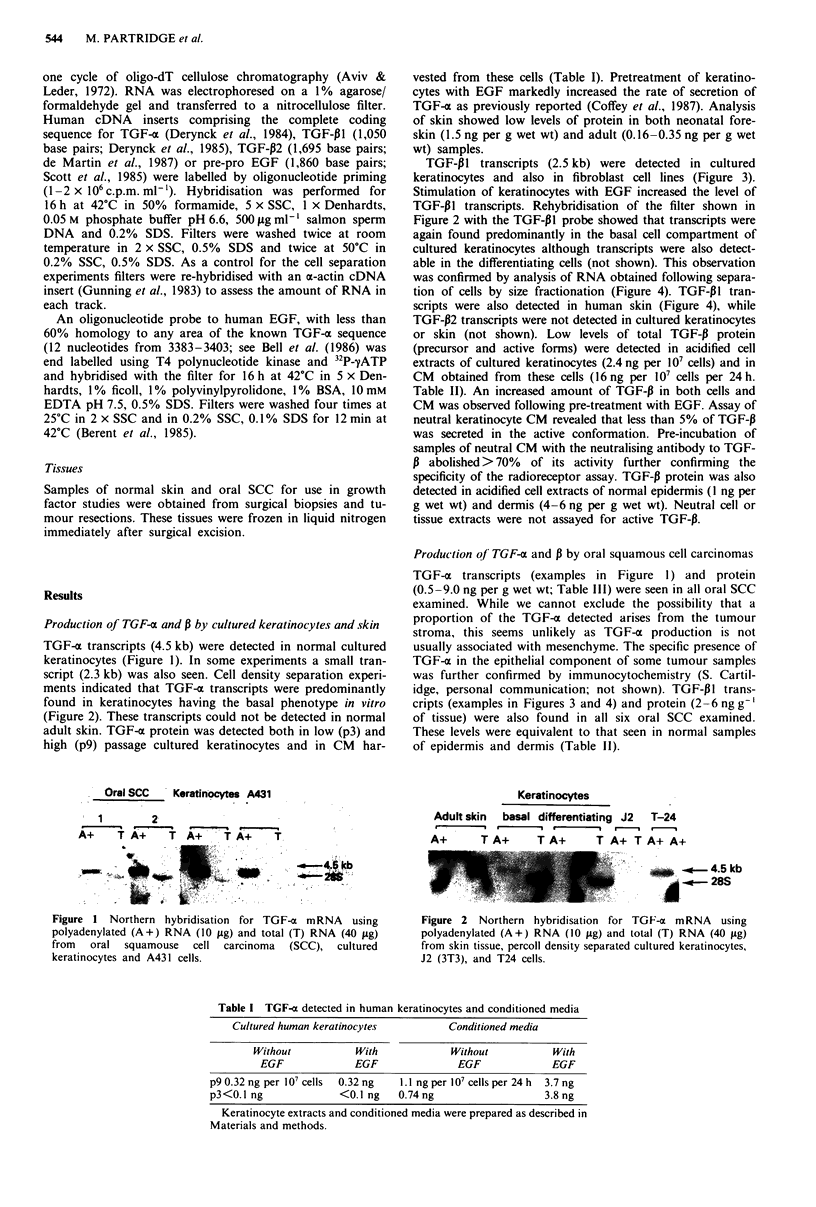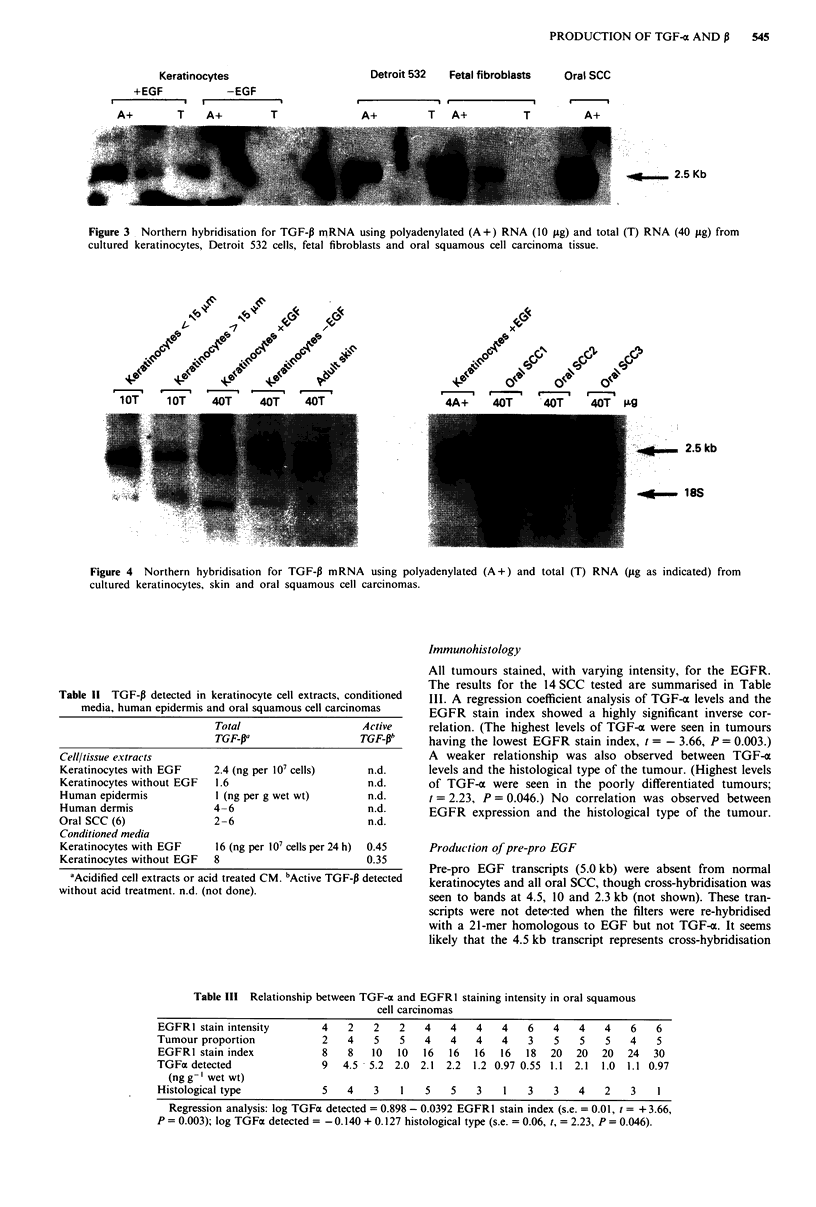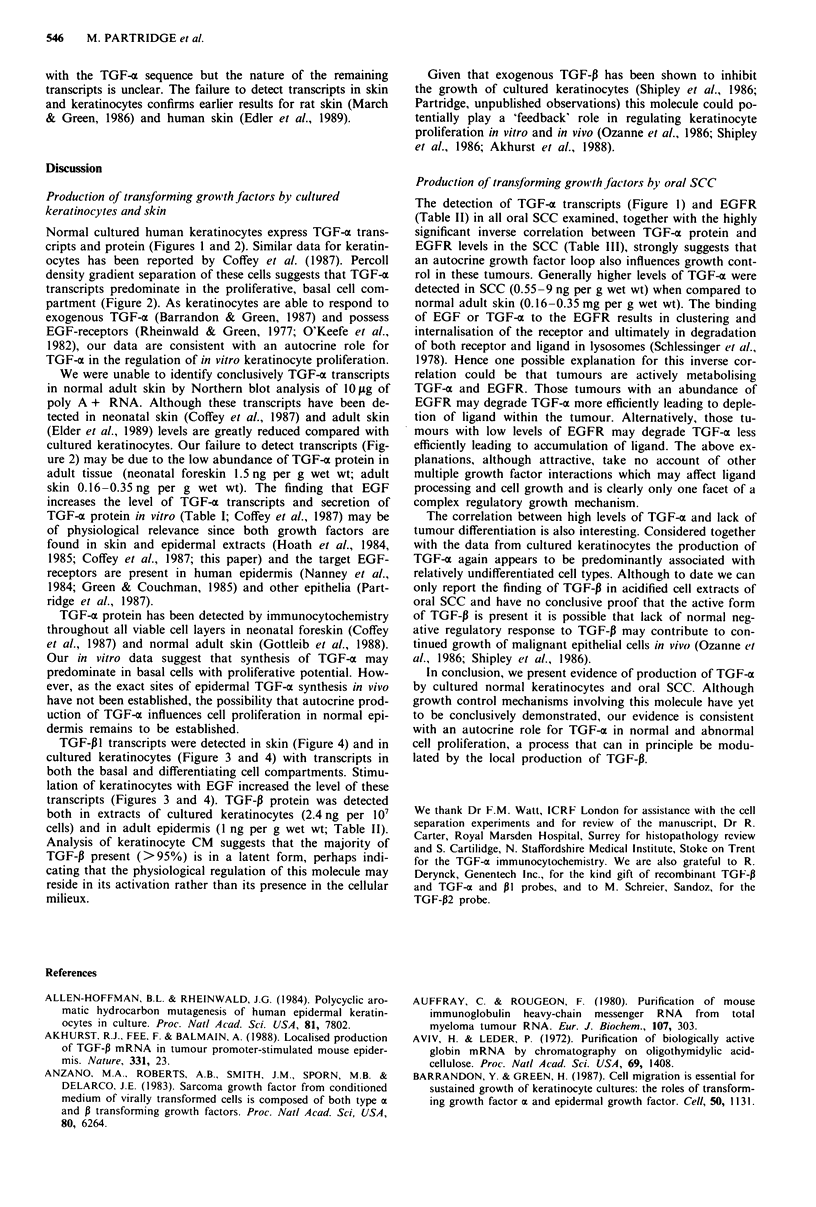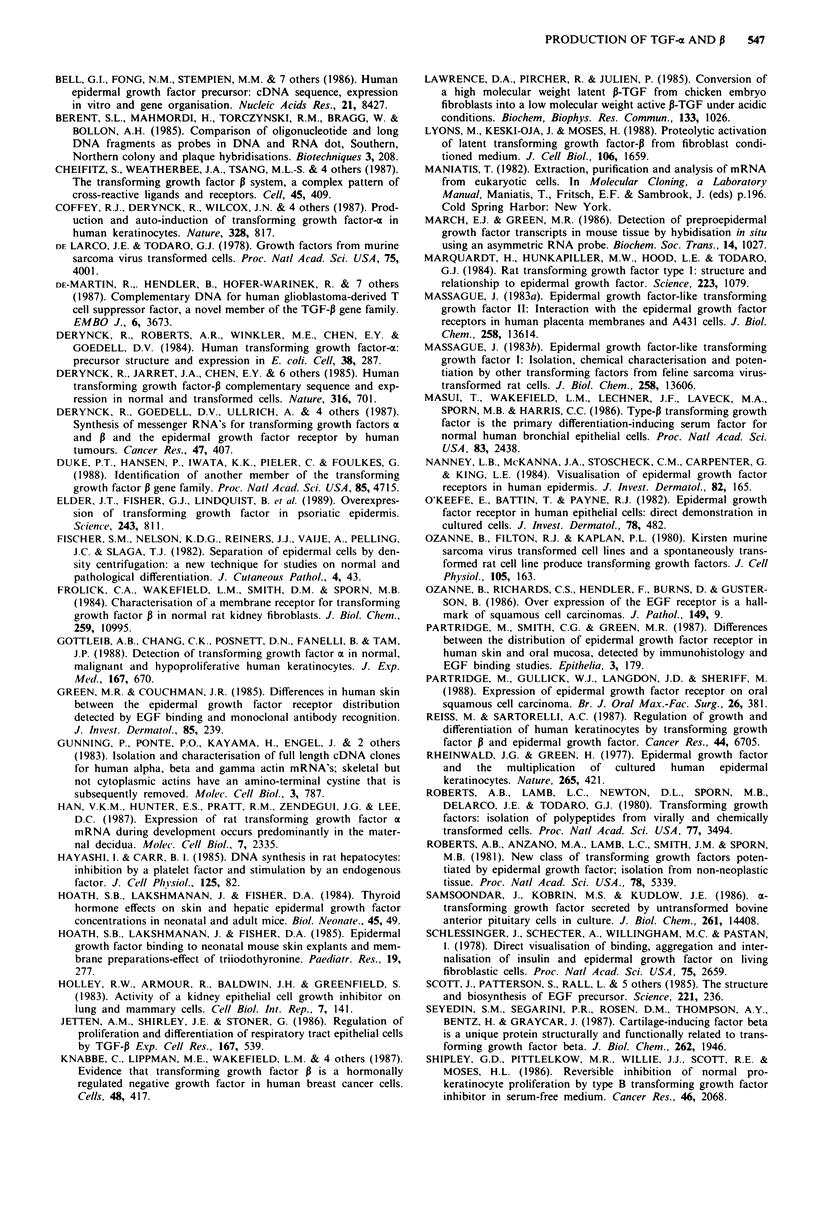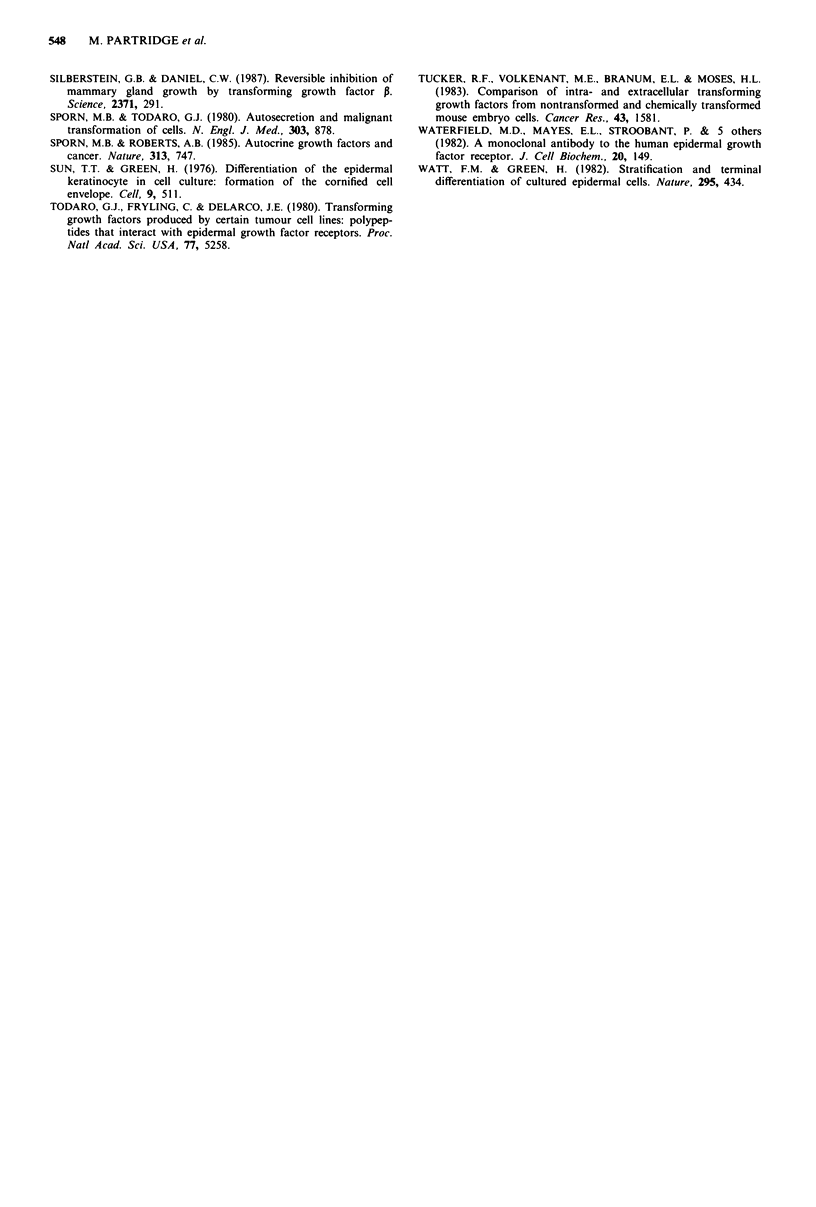# Production of TGF-alpha and TGF-beta by cultured keratinocytes, skin and oral squamous cell carcinomas--potential autocrine regulation of normal and malignant epithelial cell proliferation.

**DOI:** 10.1038/bjc.1989.310

**Published:** 1989-10

**Authors:** M. Partridge, M. R. Green, J. D. Langdon, M. Feldmann

**Affiliations:** Charing Cross Sunley Research Centre, London, UK.

## Abstract

**Images:**


Br. J. Cancer (1989), 60, 542-548 © The Macmillan Press Ltd., 1989

### Production of TGF-α and TGF-β by cultured keratinocytes, skin and oral squamous cell carcinomas – potential autocrine regulation of normal and malignant epithelial cell proliferation

M. Partridge[1], M.R. Green[2], J.D. Langdon[3] & M. Feldmann[1]

[1]*Charing Cross Sunley Research Centre, Lurgan Avenue, London W6;* [2]*Unilever Research, Colworth House, Sharnbrook, Bedford MK44 1LQ; and* [3]*Kings College Hospital, Denmark Hill, London SE5, UK.*

**Summary** Transforming growth factors have a wide range of biological activities related to cell proliferation and differentiation. In general TGF-α promotes cell proliferation while TGF-β may stimulate or inhibit proliferation depending on the cell type and growth factor environment. Cultured human keratinocytes, skin and oral squamous cell carcinomas were analysed for the presence of transcripts and protein for the transforming growth factors α & β. Both growth factors were detected in cultured keratinocytes (which have receptors for and respond to both ligands), and in medium conditioned by these cells. Additionally transcripts for TGF-α were found preferentially in the basal, proliferative compartment of cultured keratinocytes. Similarly both growth factors were detected in oral squamous cell carcinomas and a highly significant inverse correlation was found between the levels of TGF-α and the epidermal growth factor receptor in these tumours. The data for TGF-α are consistent with the existence of an autocrine growth control loop influencing cell proliferation in both a normal cell type and malignant epithelial tissues, a process that in keratinocytes and responsive squamous cell carcinomas could be modulated by TGF-β.

Transforming growth factors were originally identified and defined by their ability to induce reversible transformation of selected normal mammalian cells in culture. Exposure of anchorage-dependent cells to transforming growth factors can stimulate growth and allow cells to grow anchorage-independently and form colonies in soft agar (De Larco & Todaro, 1978; Roberts *et al.*, 1981). The identification of such factors led to the proposal of an autocrine mechanism of growth control, with transformed cells able to respond to self-produced growth factors (Sporn & Todaro, 1980; Sporn & Roberts, 1985).

Two distinct forms of transforming growth factors are now recognised, transforming growth factor alpha (TGF-α) and transforming growth factor beta (TGF-β). TGF-α is a single chain polypeptide, related to epidermal growth factor (EGF) (Marquaradt *et al.*, 1984; Derynck *et al.*, 1984), able to stimulate growth by binding to and activating the epidermal growth factor receptor (EGFR) (Todaro *et al.*, 1980; Massague, 1983*a*). TGF-α was originally described in the culture medium of retrovirally transformed fibroblasts (De Larco & Todaro, 1978; Ozanne *et al.*, 1980: Roberts *et al.*, 1980), and was subsequently detected in a number of squamous cell carcinoma (SCC) lines (Derynck *et al.*, 1987). This molecule has also been shown to be produced by a variety of normal sources including cells of the bovine anterior pituitary gland (Samsoondar *et al.*, 1986), cultured human keratinocytes, skin (Coffey *et al.*, 1987) and the maternal decidua during embryonic development (Han *et al.*, 1987). The physiological effects of TGF-α are unclear, but it can prolong the lifespan of cultured keratinocytes by stimulating lateral migration of dividing keratinocytes in expanding colonies (Barrandon & Green, 1987), suggesting a role in epidermal homeostasis.

TGF-β, a two chain polypeptide, is a member of a complex family of structurally related growth and differentiation factors. Several related forms of TGF-β have been described, including: TGF-β1, originally purified from human platelets (Derynck *et al.*, 1985) but now known to be widespread in tissues; TGF-β2, isolated from bovine bone (Seyedin *et al.*, 1987), porcine platelets (Cheifetz *et al.*, 1987) and a human glioblastoma cell line (de-Martin *et al.*, 1987); TGF-β1.2, a heterodimer of β1 and β2 subunits isolated from porcine platelets (Cheifetz *et al.*, 1987); and TGF-β3, recently isolated from a human rhabdomyosarcoma cell line (Duke *et al.*, 1988). The various forms of TGF-β bind to a set of three structurally and functionally distinct cell surface receptors (Cheifetz *et al.*, 1987).

TGF-β can be present in transformed cells which produce TGF-α (Anzano *et al.*, 1983; Massague, 1983*b*) and has also been identified in a wide range of human malignant cell lines and in normal cells of the haemopoietic and immune systems, liver and lung (Derynck *et al.*, 1987). It appears that most, if not all, cells secrete TGF-β predominantly in a latent form which, *in vitro*, after exposure to extremes of pH or proteolytic activation binds to its receptor (Lawrence *et al.*, 1985; Lyons *et al.*, 1988). The physiological mechanisms of TGF-β activation remain unclear.

Although TGF-β stimulates proliferation of some fibroblast cell types in culture (Tucker *et al.*, 1983), it has a growth inhibitory effect on epithelial cells both *in vitro* and *in vivo*, including bronchial epithelium (Holley *et al.*, 1983; Masui *et al.*, 1986; Jetten *et al.*, 1986) and mammary cells (Holley *et al.*, 1983; Knabbe *et al.*, 1987; Silberstein & Daniel, 1987), keratinocytes (Shipley *et al.*, 1986; Ozanne *et al.*, 1986; Reiss & Sartorelli, 1987), and hepatocytes (Hayashi & Carr, 1985). In contrast a number of SCC lines fail to respond to the inhibitory effects of TGF-β (Ozanne *et al.*, 1986; Shipley *et al.*, 1986). The same authors postulated that TGF-β may play a role in the regulation of normal epithelial growth, via a negative regulatory mechanism with lack of response to TGF-β leading to altered growth in carcinomas.

We have examined a series of oral SCC, cultured keratinocytes and normal skin for evidence of the production of TGF-α and β using protein and Northern blot analysis. In some cases we also examined EGF and EGFR expression. The investigation was designed to determine whether these growth factors were produced *in situ* and therefore could potentially play an autocrine role in the regulation of normal and malignant cell proliferation.

### Materials and methods

### Cell culture

Human keratinocytes derived from newborn foreskin (passage numbers 3–11) were grown in the presence of a mitomycin C treated 3T3 feeder layer (strain J2), in three parts Dulbecco's modified Eagle's medium (DMEM) and one part Ham's F12 supplemented with $1.8 \times 10^{-4}$M adenine, 10% fetal calf serum (FCS), 5 $\mu g\ ml^{-1}$ insulin, 0.4 $\mu g\ ml^{-1}$ hydrocor-

Correspondence: M. Partridge.
Received 6 January 1989; and in revised form 2 June 1989.

tisone, 8.4 ng ml$^{-1}$ cholera toxin and 10 ng ml$^{-1}$ EGF (Allen-Hoffman & Rheinwald, 1984). The medium was changed every 2 days. EGF was omitted from the culture medium in some experiments. Before the keratinocytes were harvested for cell separation experiments, RNA purification or, before collection of conditioned media, any remaining 3T3 cells were removed by vigorous washing with isotonic EDTA (Sun & Green, 1976). Human foreskin fibroblasts Detroit 532 (ATCC CRL 54) a bronchial epithelial carcinoma cell line A549 (ATCC CRL 185) and a vulval carcinoma cell line A431 (ATCC CRL 1555) were maintained in DMEM with 10% FCS. A human bladder carcinoma cell line, T-24 (ATCC-HT B-4), and human fetal fibroblasts were grown in RPMI and 10% FCS.

*Cell separation experiments*

Density gradient centrifugation of cultured keratinocytes was performed essentially as described by Fischer *et al.* (1982). Keratinocytes were filtered through a fine mesh nylon membrane to minimise clumping and aliquots of $3 \times 10^7$ cells suspended in 50% stock Percoll solution in phosphate buffered saline (PBS) (Pharmacia Fine Chemicals, Uppsala, Sweden). The suspension was centrifuged in a Sorval vertical SS 34 rotor at 16,000 r.p.m. (20,000 *g*) for 30 min at 4°C. After centrifugation two bands of concentrated cells were apparent. The upper band (density 1.049) was enriched for the larger, less dense, spinous, granular or squamous cells, the lower band (density 1.098) for the smaller spherical cells with a high nuclear-to-cytoplasmic ratio characteristic of basal cells. Keratinocytes were harvested through a thin-walled capillary tube with the aid of a peristaltic pump and washed in PBS. The reproducibility of these self-generating gradients was monitored by the use of percoll density marker beads. Keratinocytes were also separated on the basis of size by passage through a Nitex nylon monofilament screen (type HC-3-11 Tetko: Watt & Green, 1982). Cells derived from the basal layer generally passed through the membrane while the larger subrabasal cells were excluded.

*Preparation of cell, tissue extracts and conditioned medium for TGF-α protein determination*

Cell and tissue extracts were prepared by a modification of the acid/ethanol procedure (Roberts *et al.*, 1980). Frozen tissues (0.2–0.8 g) or cultured keratinocytes ($4–12 \times 10^7$ cells) were suspended (3 ml g$^{-1}$ of tissue; 1 ml per $10^7$ cells) in a solution of absolute ethanol containing 0.2 M HCl, 30 μM phenylmethylsulphonylfluoride (PMSF) and 5 μg ml$^{-1}$ pepstatin. The volume was increased one-third with distilled water and samples homogenised in a polytron (model PTA 10–35) for 30–60 s at 4°C before further PMSF was added. After overnight extraction at 4°C samples were centrifuged and re-extracted with the aqueous acid/ethanol solution.

To prepare conditioned medium (CM), near confluent dishes of keratinocytes were washed in serum-free media (SFM: DMEM and 25 mM Hepes buffer) and then washed three times at 2 h intervals. Finally cells were incubated for 24 h in SFM and CM collected. CM was dialysed against repeated changes of PBS using Spectra/por dialysis membrane, 3,500 molecular weight cut off, until the phenyl red colour was absent or greatly diminished.

Aliquots of CM (50 ml) or pooled acid/ethanol tissue extracts were trace enriched for growth factors using disposable C18 'sep-pak' reverse phase columns (Waters). Columns were prepared by washing sequentially with 3 ml of methyl alcohol, 3 ml of isopropanol and 5 ml of distilled water. Tissue or cell extracts were diluted 6–7-fold to reduce the ethanol concentration to approximately 10% and immediately loaded onto the column. The column was washed with 5 ml of PBS and bound material eluted using 50% (v/v) acetonitrile, 20 mM sodium phosphate buffer, pH 7.5. The first 500 μl of eluate was discarded, TGF-α and other bound material being eluted in the next 800 μl. Progress was conveniently followed by monitoring the elution of phenyl red added to the extracts. Acetonitrile was evaporated under a gentle stream of nitrogen and samples frozen at − 20°C unless assayed immediately. The recovery of a 5 nM TGF-α solution from the 'sep-pak' column was shown to be 80–90%. TGF-α radioimmunoassay was conducted using a commercially available kit (Biotope Inc., Seattle).

*Preparation of cell, tissue extracts and conditioned medium for TGF-β radioreceptor assay*

Cell and tissue extracts were prepared using the acid/ethanol procedure essentially as described for TGF-α. After overnight extraction at 4°C, extracts were clarified by centrifugation in a microfuge and were dialysed against repeated changes of 1 M acetic acid and concentrated by lyophilisation. Samples were reconstituted in binding buffer for the radioreceptor assay (DMEM, 0.1% BSA and 25 mM Hepes buffer). Conditioned medium was prepared by washing cells in SFM as described above and after addition of 2 mM PMSF and 0.1% BSA was clarified by centrifugation at 100,000 *g* for 30 m. Acidified CM for determination of the total amount of TGF-β present (precursor and active forms) was prepared by dialysis against repeated changes of 1 M acetic acid and concentrated by lyophilisation. Neutral aliquots of CM for determination of active TGF-β was prepared by dialysis against PBS followed by 20 mM ammonium bicarbonate prior to lyophilisation.

*TGF-β radioreceptor assay*

The $^{125}$I TGF-β radioreceptor assay was performed by a modification of the method described by Frolick *et al.* (1984). Cell and tissue extracts were assayed for TGF-β like activity by testing the ability of various concentrations of extract or CM to inhibit the binding of $^{125}$I TGF-β, (50,000 c.p.m. per well, R & D Systems Inc., Minneapolis) to A549 cells plated in 24-well cluster plates ($2 \times 10^5$ cells per well) at 4°C using the sequential assay protocol described by Assonian *et al.* (1987). Bound counts were normalised as a percentage of counts bound in control wells (typically $>$ 8000 c.p.m.) less than non-specific binding (typically $<$ 2000 c.p.m.) in the presence of an excess (10 nM) of unlabelled TGF-β. The concentration of TGF-β was calculated by reference to a standard competition curve. The radioreceptor assay does not distinguish between TGF-β1, 2, 1.2 or 3.

Neutral samples of CM were assayed in a similar fashion and in some experiments 200 μl samples were preincubated overnight at 4°C with 50 μg of neutralising antibody to TGF-β (R & D Systems Inc.). This antibody neutralises 90% of the inhibitory effect of 1 ng ml$^{-1}$ recombinant TGF-β on thymidine incorporation into mink lung epithelial cells.

*Immunohistology*

EGFR expression was investigated by means of an alkaline phosphatase (APAAP) technique using a monoclonal antibody which recognises the external domain of the EGFR designated EGFR1 (Waterfield *et al.*, 1982). The level of receptor expression was formulated into an EGFR1 stain index based on a score for both the stain intensity with the antibody and the proportion of tumour in the specimen (Partridge *et al.*, 1988). The histological type of the tumour was assigned a score value: well differentiated = 1, well/moderate = 2, moderate = 3, moderate/poor = 4, poorly differentiated = 5.

*RNA purification and hybridisation*

RNA was isolated from frozen tumours (2–5 g) or human skin (5–25 g) by homogenisation under liquid nitrogen, lysis in guanidine thiocyanate (GT) and subsequent extraction with lithium chloride/urea (Auffray & Rougeon, 1980). After removal of the feeder layer, keratinocytes were lysed in GT and RNA sedimented through caesium chloride (Maniatis, 1982). RNA was extracted from cell lines using the same technique. The polyadenylated RNA fraction was isolated by one cycle of oligo-dT cellulose chromatography (Aviv & Leder, 1972). RNA was electrophoresed on a 1% agarose/formaldehyde gel and transferred to a nitrocellulose filter. Human cDNA inserts comprising the complete coding sequence for TGF-α (Derynck *et al.*, 1984), TGF-β1 (1,050 base pairs; Derynck *et al.*, 1985), TGF-β2 (1,695 base pairs; de Martin *et al.*, 1987) or pre-pro EGF (1,860 base pairs; Scott *et al.*, 1985) were labelled by oligonucleotide priming ($1-2 \times 10^6$ c.p.m. $ml^{-1}$). Hybridisation was performed for 16 h at 42°C in 50% formamide, 5 × SSC, 1 × Denhardts, 0.05 M phosphate buffer pH 6.6, 500 μg $ml^{-1}$ salmon sperm DNA and 0.2% SDS. Filters were washed twice at room temperature in 2 × SSC, 0.5% SDS and twice at 50°C in 0.2% SSC, 0.5% SDS. As a control for the cell separation experiments filters were re-hybridised with an α-actin cDNA insert (Gunning *et al.*, 1983) to assess the amount of RNA in each track.

An oligonucleotide probe to human EGF, with less than 60% homology to any area of the known TGF-α sequence (12 nucleotides from 3383–3403; see Bell *et al.* (1986) was end labelled using T4 polynucleotide kinase and $^{32}$P-γATP and hybridised with the filter for 16 h at 42°C in 5 × Denhardts, 1% ficoll, 1% polyvinylpyrolidone, 1% BSA, 10 mM EDTA pH 7.5, 0.5% SDS. Filters were washed four times at 25°C in 2 × SSC and in 0.2% SSC, 0.1% SDS for 12 min at 42°C (Berent *et al.*, 1985).

*Tissues*

Samples of normal skin and oral SCC for use in growth factor studies were obtained from surgical biopsies and tumour resections. These tissues were frozen in liquid nitrogen immediately after surgical excision.

### Results

*Production of TGF-α and β by cultured keratinocytes and skin*

TGF-α transcripts (4.5 kb) were detected in normal cultured keratinocytes (Figure 1). In some experiments a small transcript (2.3 kb) was also seen. Cell density separation experiments indicated that TGF-α transcripts were predominantly found in keratinocytes having the basal phenotype *in vitro* (Figure 2). These transcripts could not be detected in normal adult skin. TGF-α protein was detected both in low (p3) and high (p9) passage cultured keratinocytes and in CM harvested from these cells (Table I). Pretreatment of keratinocytes with EGF markedly increased the rate of secretion of TGF-α as previously reported (Coffey *et al.*, 1987). Analysis of skin showed low levels of protein in both neonatal foreskin (1.5 ng per g wet wt) and adult (0.16–0.35 ng per g wet wt) samples.

TGF-β1 transcripts (2.5 kb) were detected in cultured keratinocytes and also in fibroblast cell lines (Figure 3). Stimulation of keratinocytes with EGF increased the level of TGF-β1 transcripts. Rehybridisation of the filter shown in Figure 2 with the TGF-β1 probe showed that transcripts were again found predominantly in the basal cell compartment of cultured keratinocytes although transcripts were also detectable in the differentiating cells (not shown). This observation was confirmed by analysis of RNA obtained following separation of cells by size fractionation (Figure 4). TGF-β1 transcripts were also detected in human skin (Figure 4), while TGF-β2 transcripts were not detected in cultured keratinocytes or skin (not shown). Low levels of total TGF-β protein (precursor and active forms) were detected in acidified cell extracts of cultured keratinocytes (2.4 ng per $10^7$ cells) and in CM obtained from these cells (16 ng per $10^7$ cells per 24 h. Table II). An increased amount of TGF-β in both cells and CM was observed following pre-treatment with EGF. Assay of neutral keratinocyte CM revealed that less than 5% of TGF-β was secreted in the active conformation. Pre-incubation of samples of neutral CM with the neutralising antibody to TGF-β abolished >70% of its activity further confirming the specificity of the radioreceptor assay. TGF-β protein was also detected in acidified cell extracts of normal epidermis (1 ng per g wet wt) and dermis (4–6 ng per g wet wt). Neutral cell or tissue extracts were not assayed for active TGF-β.

*Production of TGF-α and β by oral squamous cell carcinomas*

TGF-α transcripts (examples in Figure 1) and protein (0.5–9.0 ng per g wet wt; Table III) were seen in all oral SCC examined. While we cannot exclude the possibility that a proportion of the TGF-α detected arises from the tumour stroma, this seems unlikely as TGF-α production is not usually associated with mesenchyme. The specific presence of TGF-α in the epithelial component of some tumour samples was further confirmed by immunocytochemistry (S. Cartilidge, personal communication; not shown). TGF-β1 transcripts (examples in Figures 3 and 4) and protein (2–6 ng $g^{-1}$ of tissue) were also found in all six oral SCC examined. These levels were equivalent to that seen in normal samples of epidermis and dermis (Table II).

**Figure 1** Northern hybridisation for TGF-α mRNA using polyadenylated (A+) RNA (10 μg) and total (T) RNA (40 μg) from oral squamouse cell carcinoma (SCC), cultured keratinocytes and A431 cells.

**Figure 2** Northern hybridisation for TGF-α mRNA using polyadenylated (A+) RNA (10 μg) and total (T) RNA (40 μg) from skin tissue, percoll density separated cultured keratinocytes, J2 (3T3), and T24 cells.

**Table I** TGF-α detected in human keratinocytes and conditioned media

| Cultured human keratinocytes | | Conditioned media | |
|---|---|---|---|
| *Without EGF* | *With EGF* | *Without EGF* | *With EGF* |
| p9 0.32 ng per $10^7$ cells | 0.32 ng | 1.1 ng per $10^7$ cells per 24 h | 3.7 ng |
| p3<0.1 ng | <0.1 ng | 0.74 ng | 3.8 ng |

Keratinocyte extracts and conditioned media were prepared as described in Materials and methods.

**Figure 3** Northern hybridisation for TGF-β mRNA using polyadenylated (A+) RNA (10 μg) and total (T) RNA (40 μg) from cultured keratinocytes, Detroit 532 cells, fetal fibroblasts and oral squamous cell carcinoma tissue.

**Figure 4** Northern hybridisation for TGF-β mRNA using polyadenylated (A+) and total (T) RNA (μg as indicated) from cultured keratinocytes, skin and oral squamous cell carcinomas.

**Table II** TGF-β detected in keratinocyte cell extracts, conditioned media, human epidermis and oral squamous cell carcinomas

| | *Total TGF-β*[a] | *Active TGF-β*[b] |
|---|---|---|
| *Cell/tissue extracts* | | |
| Keratinocytes with EGF | 2.4 (ng per $10^7$ cells) | n.d. |
| Keratinocytes without EGF | 1.6 | n.d. |
| Human epidermis | 1 (ng per g wet wt) | n.d. |
| Human dermis | 4–6 | n.d. |
| Oral SCC (6) | 2–6 | n.d. |
| *Conditioned media* | | |
| Keratinocytes with EGF | 16 (ng per $10^7$ cells per 24 h) | 0.45 |
| Keratinocytes without EGF | 8 | 0.35 |

[a]Acidified cell extracts or acid treated CM. [b]Active TGF-β detected without acid treatment. n.d. (not done).

### *Immunohistology*

All tumours stained, with varying intensity, for the EGFR. The results for the 14 SCC tested are summarised in Table III. A regression coefficient analysis of TGF-α levels and the EGFR stain index showed a highly significant inverse correlation. (The highest levels of TGF-α were seen in tumours having the lowest EGFR stain index, $t = -3.66$, $P = 0.003$.) A weaker relationship was also observed between TGF-α levels and the histological type of the tumour. (Highest levels of TGF-α were seen in the poorly differentiated tumours; $t = 2.23$, $P = 0.046$.) No correlation was observed between EGFR expression and the histological type of the tumour.

### *Production of pre-pro EGF*

Pre-pro EGF transcripts (5.0 kb) were absent from normal keratinocytes and all oral SCC, though cross-hybridisation was seen to bands at 4.5, 10 and 2.3 kb (not shown). These transcripts were not detected when the filters were re-hybridised with a 21-mer homologous to EGF but not TGF-α. It seems likely that the 4.5 kb transcript represents cross-hybridisation

**Table III** Relationship between TGF-α and EGFR1 staining intensity in oral squamous cell carcinomas

| | | | | | | | | | | | | | | |
|---|---|---|---|---|---|---|---|---|---|---|---|---|---|---|
| EGFR1 stain intensity | 4 | 2 | 2 | 2 | 4 | 4 | 4 | 4 | 6 | 4 | 4 | 4 | 6 | 6 |
| Tumour proportion | 2 | 4 | 5 | 5 | 4 | 4 | 4 | 4 | 3 | 5 | 5 | 5 | 4 | 5 |
| EGFR1 stain index | 8 | 8 | 10 | 10 | 16 | 16 | 16 | 16 | 18 | 20 | 20 | 20 | 24 | 30 |
| TGFα detected ($ng\ g^{-1}$ wet wt) | 9 | 4.5 | 5.2 | 2.0 | 2.1 | 2.2 | 1.2 | 0.97 | 0.55 | 1.1 | 2.1 | 1.0 | 1.1 | 0.97 |
| Histological type | 5 | 4 | 3 | 1 | 5 | 5 | 3 | 1 | 3 | 3 | 4 | 2 | 3 | 1 |

Regression analysis: log TGFα detected = 0.898 − 0.0392 EGFR1 stain index (s.e. = 0.01, $t = +3.66$, $P = 0.003$); log TGFα detected = −0.140 + 0.127 histological type (s.e. = 0.06, $t_s = 2.23$, $P = 0.046$).

with the TGF-α sequence but the nature of the remaining transcripts is unclear. The failure to detect transcripts in skin and keratinocytes confirms earlier results for rat skin (March & Green, 1986) and human skin (Edler *et al.*, 1989).

### Discussion

*Production of transforming growth factors by cultured keratinocytes and skin*

Normal cultured human keratinocytes express TGF-α transcripts and protein (Figures 1 and 2). Similar data for keratinocytes has been reported by Coffey *et al.* (1987). Percoll density gradient separation of these cells suggests that TGF-α transcripts predominate in the proliferative, basal cell compartment (Figure 2). As keratinocytes are able to respond to exogenous TGF-α (Barrandon & Green, 1987) and possess EGF-receptors (Rheinwald & Green, 1977; O'Keefe *et al.*, 1982), our data are consistent with an autocrine role for TGF-α in the regulation of *in vitro* keratinocyte proliferation.

We were unable to identify conclusively TGF-α transcripts in normal adult skin by Northern blot analysis of 10 μg of poly A + RNA. Although these transcripts have been detected in neonatal skin (Coffey *et al.*, 1987) and adult skin (Elder *et al.*, 1989) levels are greatly reduced compared with cultured keratinocytes. Our failure to detect transcripts (Figure 2) may be due to the low abundance of TGF-α protein in adult tissue (neonatal foreskin 1.5 ng per g wet wt; adult skin 0.16–0.35 ng per g wet wt). The finding that EGF increases the level of TGF-α transcripts and secretion of TGF-α protein *in vitro* (Table I; Coffey *et al.*, 1987) may be of physiological relevance since both growth factors are found in skin and epidermal extracts (Hoath *et al.*, 1984, 1985; Coffey *et al.*, 1987; this paper) and the target EGF-receptors are present in human epidermis (Nanney *et al.*, 1984; Green & Couchman, 1985) and other epithelia (Partridge *et al.*, 1987).

TGF-α protein has been detected by immunocytochemistry throughout all viable cell layers in neonatal foreskin (Coffey *et al.*, 1987) and normal adult skin (Gottleib *et al.*, 1988). Our *in vitro* data suggest that synthesis of TGF-α may predominate in basal cells with proliferative potential. However, as the exact sites of epidermal TGF-α synthesis *in vivo* have not been established, the possibility that autocrine production of TGF-α influences cell proliferation in normal epidermis remains to be established.

TGF-β1 transcripts were detected in skin (Figure 4) and in cultured keratinocytes (Figure 3 and 4) with transcripts in both the basal and differentiating cell compartments. Stimulation of keratinocytes with EGF increased the level of these transcripts (Figures 3 and 4). TGF-β protein was detected both in extracts of cultured keratinocytes (2.4 ng per $10^7$ cells) and in adult epidermis (1 ng per g wet wt; Table II). Analysis of keratinocyte CM suggests that the majority of TGF-β present (>95%) is in a latent form, perhaps indicating that the physiological regulation of this molecule may reside in its activation rather than its presence in the cellular milieux.

Given that exogenous TGF-β has been shown to inhibit the growth of cultured keratinocytes (Shipley *et al.*, 1986; Partridge, unpublished observations) this molecule could potentially play a 'feedback' role in regulating keratinocyte proliferation *in vitro* and *in vivo* (Ozanne *et al.*, 1986; Shipley *et al.*, 1986; Akhurst *et al.*, 1988).

*Production of transforming growth factors by oral SCC*

The detection of TGF-α transcripts (Figure 1) and EGFR (Table II) in all oral SCC examined, together with the highly significant inverse correlation between TGF-α protein and EGFR levels in the SCC (Table III), strongly suggests that an autocrine growth factor loop also influences growth control in these tumours. Generally higher levels of TGF-α were detected in SCC (0.55–9 ng per g wet wt) when compared to normal adult skin (0.16–0.35 mg per g wet wt). The binding of EGF or TGF-α to the EGFR results in clustering and internalisation of the receptor and ultimately in degradation of both receptor and ligand in lysosomes (Schlessinger *et al.*, 1978). Hence one possible explanation for this inverse correlation could be that tumours are actively metabolising TGF-α and EGFR. Those tumours with an abundance of EGFR may degrade TGF-α more efficiently leading to depletion of ligand within the tumour. Alternatively, those tumours with low levels of EGFR may degrade TGF-α less efficiently leading to accumulation of ligand. The above explanations, although attractive, take no account of other multiple growth factor interactions which may affect ligand processing and cell growth and is clearly only one facet of a complex regulatory growth mechanism.

The correlation between high levels of TGF-α and lack of tumour differentiation is also interesting. Considered together with the data from cultured keratinocytes the production of TGF-α again appears to be predominantly associated with relatively undifferentiated cell types. Although to date we can only report the finding of TGF-β in acidified cell extracts of oral SCC and have no conclusive proof that the active form of TGF-β is present it is possible that lack of normal negative regulatory response to TGF-β may contribute to continued growth of malignant epithelial cells *in vivo* (Ozanne *et al.*, 1986; Shipley *et al.*, 1986).

In conclusion, we present evidence of production of TGF-α by cultured normal keratinocytes and oral SCC. Although growth control mechanisms involving this molecule have yet to be conclusively demonstrated, our evidence is consistent with an autocrine role for TGF-α in normal and abnormal cell proliferation, a process that can in principle be modulated by the local production of TGF-β.

We thank Dr F.M. Watt, ICRF London for assistance with the cell separation experiments and for review of the manuscript, Dr R. Carter, Royal Marsden Hospital, Surrey for histopathology review and S. Cartilidge, N. Staffordshire Medical Institute, Stoke on Trent for the TGF-α immunocytochemistry. We are also grateful to R. Derynck, Genentech Inc., for the kind gift of recombinant TGF-β and TGF-α and β1 probes, and to M. Schreier, Sandoz, for the TGF-β2 probe.

### References

ALLEN-HOFFMAN, B.L. & RHEINWALD, J.G. (1984). Polycyclic aromatic hydrocarbon mutagenesis of human epidermal keratinocytes in culture. *Proc. Natl Acad. Sci. USA*, **81**, 7802.

AKHURST, R.J., FEE, F. & BALMAIN, A. (1988). Localised production of TGF-β mRNA in tumour promoter-stimulated mouse epidermis. *Nature*, **331**, 23.

ANZANO, M.A., ROBERTS, A.B., SMITH, J.M., SPORN, M.B. & DELARCO, J.E. (1983). Sarcoma growth factor from conditioned medium of virally transformed cells is composed of both type α and β transforming growth factors. *Proc. Natl Acad. Sci, USA*, **80**, 6264.

AUFFRAY, C. & ROUGEON, F. (1980). Purification of mouse immunoglobulin heavy-chain messenger RNA from total myeloma tumour RNA. *Eur. J. Biochem.*, **107**, 303.

AVIV, H. & LEDER, P. (1972). Purification of biologically active globin mRNA by chromatography on oligothymidylic acid-cellulose. *Proc. Natl Acad. Sci. USA*, **69**, 1408.

BARRANDON, Y. & GREEN, H. (1987). Cell migration is essential for sustained growth of keratinocyte cultures: the roles of transforming growth factor α and epidermal growth factor. *Cell*, **50**, 1131.

BELL, G.I., FONG, N.M., STEMPIEN, M.M. & 7 others (1986). Human epidermal growth factor precursor: cDNA sequence, expression in vitro and gene organisation. *Nucleic Acids Res.*, **21**, 8427.

BERENT, S.L., MAHMORDI, H., TORCZYNSKI, R.M., BRAGG, W. & BOLLON, A.H. (1985). Comparison of oligonucleotide and long DNA fragments as probes in DNA and RNA dot, Southern, Northern colony and plaque hybridisations. *Biotechniques* **3**, 208.

CHEIFITZ, S., WEATHERBEE, J.A., TSANG, M.L.-S. & 4 others (1987). The transforming growth factor β system, a complex pattern of cross-reactive ligands and receptors. *Cell*, **45**, 409.

COFFEY, R.J., DERYNCK, R., WILCOX, J.N. & 4 others (1987). Production and auto-induction of transforming growth factor-α in human keratinocytes. *Nature*, **328**, 817.

DE LARCO, J.E. & TODARO, G.J. (1978). Growth factors from murine sarcoma virus transformed cells. *Proc. Natl Acad. Sci. USA*, **75**, 4001.

DE-MARTIN, R., HENDLER, B., HOFER-WARINEK, R. & 7 others (1987). Complementary DNA for human glioblastoma-derived T cell suppressor factor, a novel member of the TGF-β gene family. *EMBO J.*, **6**, 3673.

DERYNCK, R., ROBERTS, A.R., WINKLER, M.E., CHEN, E.Y. & GOEDELL, D.V. (1984). Human transforming growth factor-α: precursor structure and expression in *E. coli*. *Cell*, **38**, 287.

DERYNCK, R., JARRET, J.A., CHEN, E.Y. & 6 others (1985). Human transforming growth factor-β complementary sequence and expression in normal and transformed cells. *Nature*, **316**, 701.

DERYNCK, R., GOEDELL, D.V., ULLRICH, A. & 4 others (1987). Synthesis of messenger RNA's for transforming growth factors α and β and the epidermal growth factor receptor by human tumours. *Cancer Res.*, **47**, 407.

DUKE, P.T., HANSEN, P., IWATA, K.K., PIELER, C. & FOULKES, G. (1988). Identification of another member of the transforming growth factor β gene family. *Proc. Natl Acad. Sci. USA*, **85**, 4715.

ELDER, J.T., FISHER, G.J., LINDQUIST, B. *et al.* (1989). Overexpression of transforming growth factor in psoriatic epidermis. *Science*, **243**, 811.

FISCHER, S.M., NELSON, K.D.G., REINERS, J.J., VAIJE, A., PELLING, J.C. & SLAGA, T.J. (1982). Separation of epidermal cells by density centrifugation: a new technique for studies on normal and pathological differentiation. *J. Cutaneous Pathol.*, **4**, 43.

FROLICK, C.A., WAKEFIELD, L.M., SMITH, D.M. & SPORN, M.B. (1984). Characterisation of a membrane receptor for transforming growth factor β in normal rat kidney fibroblasts. *J. Biol. Chem.*, **259**, 10995.

GOTTLEIB, A.B., CHANG, C.K., POSNETT, D.N., FANELLI, B. & TAM, J.P. (1988). Detection of transforming growth factor α in normal, malignant and hypoproliferative human keratinocytes. *J. Exp. Med.*, **167**, 670.

GREEN, M.R. & COUCHMAN, J.R. (1985). Differences in human skin between the epidermal growth factor receptor distribution detected by EGF binding and monoclonal antibody recognition. *J. Invest. Dermatol.*, **85**, 239.

GUNNING, P., PONTE, P.O., KAYAMA, H., ENGEL, J. & 2 others (1983). Isolation and characterisation of full length cDNA clones for human alpha, beta and gamma actin mRNA's; skeletal but not cytoplasmic actins have an amino-terminal cystine that is subsequently removed. *Molec. Cell Biol.*, **3**, 787.

HAN, V.K.M., HUNTER, E.S., PRATT, R.M., ZENDEGUI, J.G. & LEE, D.C. (1987). Expression of rat transforming growth factor α mRNA during development occurs predominantly in the maternal decidua. *Molec. Cell Biol.*, **7**, 2335.

HAYASHI, I. & CARR, B. I. (1985). DNA synthesis in rat hepatocytes: inhibition by a platelet factor and stimulation by an endogenous factor. *J. Cell Physiol.*, **125**, 82.

HOATH, S.B., LAKSHMANAN, J. & FISHER, D.A. (1984). Thyroid hormone effects on skin and hepatic epidermal growth factor concentrations in neonatal and adult mice. *Biol. Neonate.*, **45**, 49.

HOATH, S.B., LAKSHMANAN, J. & FISHER, D.A. (1985). Epidermal growth factor binding to neonatal mouse skin explants and membrane preparations-effect of triiodothyronine. *Paediatr. Res.*, **19**, 277.

HOLLEY, R.W., ARMOUR, R., BALDWIN, J.H. & GREENFIELD, S. (1983). Activity of a kidney epithelial cell growth inhibitor on lung and mammary cells. *Cell Biol. Int. Rep.*, **7**, 141.

JETTEN, A.M., SHIRLEY, J.E. & STONER, G. (1986). Regulation of proliferation and differentiation of respiratory tract epithelial cells by TGF-β *Exp. Cell Res.*, **167**, 539.

KNABBE, C., LIPPMAN, M.E., WAKEFIELD, L.M. & 4 others (1987). Evidence that transforming growth factor β is a hormonally regulated negative growth factor in human breast cancer cells. *Cells*, **48**, 417.

LAWRENCE, D.A., PIRCHER, R. & JULIEN, P. (1985). Conversion of a high molecular weight latent β-TGF from chicken embryo fibroblasts into a low molecular weight active β-TGF under acidic conditions. *Biochem, Biophys. Res. Commun.*, **133**, 1026.

LYONS, M., KESKI-OJA, J. & MOSES, H. (1988). Proteolytic activation of latent transforming growth factor-β from fibroblast conditioned medium. *J. Cell Biol.*, **106**, 1659.

MANIATIS, T. (1982). Extraction, purification and analysis of mRNA from eukaryotic cells. In *Molecular Cloning, a Laboratory Manual*, Maniatis, T., Fritsch, E.F. & Sambrook, J. (eds) p.196. Cold Spring Harbor: New York.

MARCH, E.J. & GREEN, M.R. (1986). Detection of preproepidermal growth factor transcripts in mouse tissue by hybidisation *in situ* using an asymmetric RNA probe. *Biochem. Soc. Trans.*, **14**, 1027.

MARQUARDT, H., HUNKAPILLER, M.W., HOOD, L.E. & TODARO, G.J. (1984). Rat transforming growth factor type 1: structure and relationship to epidermal growth factor. *Science*, **223**, 1079.

MASSAGUE, J. (1983*a*). Epidermal growth factor-like transforming growth factor II: Interaction with the epidermal growth factor receptors in human placenta membranes and A431 cells. *J. Biol. Chem.*, **258**, 13614.

MASSAGUE, J. (1983*b*). Epidermal growth factor-like transforming growth factor I: Isolation, chemical characterisation and potentiation by other transforming factors from feline sarcoma virus-transformed rat cells. *J. Biol. Chem.*, **258**, 13606.

MASUI, T., WAKEFIELD, L.M., LECHNER, J.F., LAVECK, M.A., SPORN, M.B. & HARRIS, C.C. (1986). Type-β transforming growth factor is the primary differentiation-inducing serum factor for normal human bronchial epithelial cells. *Proc. Natl Acad. Sci. USA*, **83**, 2438.

NANNEY, L.B., McKANNA, J.A., STOSCHECK, C.M., CARPENTER, G. & KING, L.E. (1984). Visualisation of epidermal growth factor receptors in human epidermis. *J. Invest. Dermatol.*, **82**, 165.

O'KEEFE, E., BATTIN, T. & PAYNE, R.J. (1982). Epidermal growth factor receptor in human epithelial cells: direct demonstration in cultured cells. *J. Invest. Dermatol.*, **78**, 482.

OZANNE, B., FILTON, R.J. & KAPLAN, P.L. (1980). Kirsten murine sarcoma virus transformed cell lines and a spontaneously transformed rat cell line produce transforming growth factors. *J. Cell Physiol.*, **105**, 163.

OZANNE, B., RICHARDS, C.S., HENDLER, F., BURNS, D. & GUSTERSON, B. (1986). Over expression of the EGF receptor is a hallmark of squamous cell carcinomas. *J. Pathol.*, **149**, 9.

PARTRIDGE, M., SMITH, C.G. & GREEN, M.R. (1987). Differences between the distribution of epidermal growth factor receptor in human skin and oral mucosa, detected by immunohistology and EGF binding studies. *Epithelia*, **3**, 179.

PARTRIDGE, M., GULLICK, W.J., LANGDON, J.D. & SHERIFF, M. (1988). Expression of epidermal growth factor receptor on oral squamous cell carcinoma. *Br. J. Oral Max.-Fac. Surg.*, **26**, 381.

REISS, M. & SARTORELLI, A.C. (1987). Regulation of growth and differentiation of human keratinocytes by transforming growth factor β and epidermal growth factor. *Cancer Res.*, **44**, 6705.

RHEINWALD, J.G. & GREEN, H. (1977). Epidermal growth factor and the multiplication of cultured human epidermal keratinocytes. *Nature*, **265**, 421.

ROBERTS, A.B., LAMB, L.C., NEWTON, D.L., SPORN, M.B., DELARCO, J.E. & TODARO, G.J. (1980). Transforming growth factors: isolation of polypeptides from virally and chemically transformed cells. *Proc. Natl Acad. Sci. USA*, **77**, 3494.

ROBERTS, A.B., ANZANO, M.A., LAMB, L.C., SMITH, J.M. & SPORN, M.B. (1981). New class of transforming growth factors potentiated by epidermal growth factor; isolation from non-neoplastic tissue. *Proc. Natl Acad. Sci. USA.*, **78**, 5339.

SAMSOONDAR, J., KOBRIN, M.S. & KUDLOW, J.E. (1986). α-transforming growth factor secreted by untransformed bovine anterior pituitary cells in culture. *J. Biol. Chem.*, **261**, 14408.

SCHLESSINGER, J., SCHECTER, A., WILLINGHAM, M.C. & PASTAN, I. (1978). Direct visualisation of binding, aggregation and internalisation of insulin and epidermal growth factor on living fibroblastic cells. *Proc. Natl Acad. Sci. USA*, **75**, 2659.

SCOTT, J., PATTERSON, S., RALL, L. & 5 others (1985). The structure and biosynthesis of EGF precursor. *Science*, **221**, 236.

SEYEDIN, S.M., SEGARINI, P.R., ROSEN, D.M., THOMPSON, A.Y., BENTZ, H. & GRAYCAR, J. (1987). Cartilage-inducing factor beta is a unique protein structurally and functionally related to transforming growth factor beta. *J. Biol. Chem.*, **262**, 1946.

SHIPLEY, G.D., PITTLELKOW, M.R., WILLIE, J.J., SCOTT, R.E. & MOSES, H.L. (1986). Reversible inhibition of normal prokeratinocyte proliferation by type B transforming growth factor inhibitor in serum-free medium. *Cancer Res.*, **46**, 2068.

SILBERSTEIN, G.B. & DANIEL, C.W. (1987). Reversible inhibition of mammary gland growth by transforming growth factor β. *Science*, **2371**, 291.

SPORN, M.B. & TODARO, G.J. (1980). Autosecretion and malignant transformation of cells. *N. Engl. J. Med.*, **303**, 878.

SPORN, M.B. & ROBERTS, A.B. (1985). Autocrine growth factors and cancer. *Nature*, **313**, 747.

SUN, T.T. & GREEN, H. (1976). Differentiation of the epidermal keratinocyte in cell culture: formation of the cornified cell envelope. *Cell*, **9**, 511.

TODARO, G.J., FRYLING, C. & DELARCO, J.E. (1980). Transforming growth factors produced by certain tumour cell lines: polypeptides that interact with epidermal growth factor receptors. *Proc. Natl Acad. Sci. USA*, **77**, 5258.

TUCKER, R.F., VOLKENANT, M.E., BRANUM, E.L. & MOSES, H.L. (1983). Comparison of intra- and extracellular transforming growth factors from nontransformed and chemically transformed mouse embryo cells. *Cancer Res.*, **43**, 1581.

WATERFIELD, M.D., MAYES, E.L., STROOBANT, P. & 5 others (1982). A monoclonal antibody to the human epidermal growth factor receptor. *J. Cell Biochem.*, **20**, 149.

WATT, F.M. & GREEN, H. (1982). Stratification and terminal differentiation of cultured epidermal cells. *Nature*, **295**, 434.